# A project report on the switch from in-class to online tuition in the preparatory course for the knowledge examination for foreign physicians – guidelines for the organization of the online summer semester 2020 at the faculty of medicine of the Westfälische Wilhelms-Universität of Münster

**DOI:** 10.3205/zma001484

**Published:** 2021-06-15

**Authors:** Zornitsa Shomanova, Helmut Ahrens, Tanja dos Santos, Janina Sensmeier, Rahel Kurpat, Maike Schnase, Kemal Yildirim, Bernhard Marschall

**Affiliations:** 1 University Hospital Münster, Department of Cardiology I, Coronary and Peripheral Vascular Disease, Heart Failure, Münster, Germany; 2 Westfälische Wilhelms-Universität Münster, Institute of medical education, Münster, Germany

**Keywords:** online course, knowledge examination, foreign physicians

## Abstract

**Introduction:** In Germany, foreign physicians are a fixed component of the medical profession. According to the German Medical Licensure Act, physicians having completed their qualification in another country are required to pass a knowledge examination which falls within the competence of examination offices or the regional governments.

**Project outline: **The preparatory course consists of 10 modules. On Fridays, individual cases are discussed in small groups and specific examination techniques are trained. On Saturdays, illnesses are simulated by simulated patients. After each encounter, faculty experts, psychologists and peer group members provide the participants with 360° feedback.

Due to the COVID-19 pandemic, the course which had been established 2 years beforehand has now been switched to an online class within one week. Friday units were visualized in power-point presentations and tutorial videos were discussed. On Saturdays, the cases were simulated by simulated patients and transmitted via a telemedicine platform.

**Results: **The course could be conducted without interruptions (75 hours of in-class tuition and 75 hours of online tuition). In the oral evaluation the participants criticized telemedicine as a medium for imparting of practical skills. 7/22 (32%) of the participants underwent the knowledge examination and 6/7 (86%) of them passed it (versus 18/19 of the participants of in-class tuition (95%)).

**Discussion:** There was a clear preference for in-class tuition. It was noted that the telemedical setting entailed some restrictions. However, the switch to online classes did not affect the pass rate.

**Conclusion:** The switch from in-class to online units was feasible. The gained insights were taken into account when conceiving the online semester at our faculty and especially the tuition with the support of simulated patients.

## Zusammenfassung

**Einleitung: **Ausländische ÄrztInnen sind in Deutschland fester Bestandteil der Ärzteschaft. ÄrztInnen mit einer Ausbildung aus einem Drittstaat müssen laut der Approbationsordnung für Ärzte eine Kenntnisprüfung ablegen, für die die Landesprüfungsämter oder die Bezirksregierungen zuständig sind.

**Projektbeschreibung: **Der Vorbereitungskurs besteht aus 10 Modulen. Freitags werden Fälle in Kleingruppen besprochen und fachspezifische Untersuchungstechniken geübt. Samstags werden mit SimulationspatientInnen bestimmte Krankheiten simuliert. Nach der Interaktion bekommen die TeilnehmerInnen ein 360°-Feedback von FachdozentInnen, PsychologInnen und KollegInnen aus der Gruppe.

Durch die Corona-Pandemie wurde der schon seit 2 Jahren bestehende Kurs innerhalb von einer Woche auf ein Onlineformat umgestellt. Die Freitagseinheiten wurden mit Power-Point visualisiert und Lernvideos besprochen. Samstags wurden die Fälle mit SimulationspatientInnen telemedizinisch simuliert.

**Ergebnisse: **Der Kurs konnte ohne Unterbrechung durchgeführt werden (75 Stunden als Präsenz-, 75 Stunden als Online-Unterricht). In der mündlichen Evaluation der TeilnehmerInnen wurde die telemedizinische Vermittlung von praktischen Fertigkeiten bemängelt. 7/22 (32%) der TeilnehmerInnen haben an der Kenntnisprüfung teilgenommen, 6/7 (86%) haben die Prüfung bestanden (in Präsenzunterricht 18/19 (95%)).

**Diskussion: **Der Präsenzunterricht wurde eindeutig präferiert. Es wurden Einschränkungen durch das telemedizinische Setting festgestellt. Die Bestehungsquote hat sich durch die Umstellung nicht verändert.

**Schlussfolgerung: **Die Umstellung von Präsenzeinheiten auf Online war möglich. Die Erfahrungen flossen in die Entwicklung der Online-Semester an unserer Fakultät ein, insbesondere der Unterricht mit den SimulationspatientInnen.

## 1. Introduction

In Germany, a shortage of physicians has been deplored since 2002 [1]. As a result of the age structure of the members of the medical profession and the increased loss of younger physicians from patient care, hospitals suffer from a high number of vacant medical positions [https://www.kbv.de/html/5724.php]. Politics currently discuss different mitigating strategies to fill the gaps such as country doctor ratios, different selection procedures for medical studies, financial support for continuing education in general medicine [https://www.gesetze-im-internet.de/sgb_5/__75a.html]. Foreign physicians are part of this strategy. According to the latest statistics the Federal Medical Council issued in 2019 there are 58,168 foreign physicians working in Germany hailing from all over the world [2]. This amounts to approx. 10% of the whole medical profession in Germany, with the numbers rising steadily each year [2].

In order to be able to practice medicine in the German healthcare system foreign physicians require a valid Medical Licensure. In the case of physicians hailing from member states of the European Union (EU) their Medical Licensure is recognized within the framework of the EU-provisions regarding the recognition of professional qualifications [https://ec.europa.eu/growth/single-market/services/free-movement-professionals/policy/legislation/].

In the case of physicians originating from countries that are not member states of the EU/ European Economic Area (EEA) a different procedure is used; their qualification certificates are verified by the licensure authorities which require physicians to take a so-called knowledge examination when it is established that the medical training in the non-European country in which they acquired their qualification differs significantly from that in Germany. This examination is a sort of verification of the equivalence of their knowledge to that required in the third part of the medical examination in the German medical studies. According to section 12 para 3 of the Federal Medical Code the competent body under state law is responsible for the conduct of this knowledge examination [https://www.gesetze-im-internet.de/b_o/BJNR018570961.html] – normally, it is the state examination offices and regional governments which are responsible for its organization and implementation. Having successfully passed the examination, physicians are granted the German licensure including permission to carry out their medical profession in Germany. The knowledge examination may be repeated twice. Having failed the examination both times foreign physicians permanently lose their chance to work in Germany as a physician [http://www.gesetze-im-internet.de/_appro_2002/BJNR240500002.html]. Therefore, apart from the verification of the level of expertise the knowledge examination also has existential consequences for the candidates.

According to the German Medical Licensure Act the knowledge examination is an oral and practical test including a case presentation [http://www.gesetze-im-internet.de/_appro_2002/BJNR240500002.html]. In the practical part, physicians are assessed at the patient’s bedside in view of their evaluation of the patient’s medical history, their physical examination and their composition of a discharge letter. In the second part of the examination an examination board consisting of four examiners evaluates the physicians’ specialist knowledge. The examination subjects are internal medicine and surgery and the questions additionally include aspects of the subjects emergency medicine, clinical pharmacology/pharmacotherapy, imaging methods, radioprotection and legal issues of the medical profession [http://www.gesetze-im-internet.de/_appro_2002/BJNR240500002.html].

In order to prepare the physicians for the knowledge examination and their profession in the medical system in the best way possible we at the Faculty of Medicine of the Westfälische Wilhelms-Universität of Münster developed the project “Competence-based Medical Qualification – Preparatory Course for the Knowledge Examination with Particular Focus on Rural Areas – Komed-Q”. It is a subproject of the support program “Integration by Qualification” (IQ-Network, NRW) and is promoted by the Federal Ministry of Labor and Social Affairs (BMAS) and the European Social Funds (ESF (Art. 8 (EG) Nr. 1828/2006)).

## 2. Project outline

The project’s content consists of three components – case-based learning with self-study E-learning, physical examination techniques as well as physician-patient communication training. The learning theory of the course is based on the known Inverted Classroom Concept [3]. Apart from consolidating of expert knowledge in the practice the project focuses on communication between physicians and patients and on the targeted coaching of participants. The self-study of theoretical expert knowledge is supported by an ILIAS-based E-learning tool which contains a selection of educational articles and tutorials. In the course the acquired knowledge is consolidated on the basis of case reviews. The already mastered physical examination techniques are adapted to the legal and medical standards applying in Germany. Cultural peculiarities as well as specifics of the medical healthcare system are practiced on simulated patients in a secure environment. The course is held on ten weekends namely Friday and Saturday with obligatory attendance.

### 2.1. Detailed course description under previous circumstances

At the beginning of the course applicants submit themselves to a competence assessment procedure consisting of a written test (60 multiple-choice questions pertaining to the subjects relevant to the knowledge examination) and a practical parcours test consisting of six practice-oriented, clinically relevant medical cases (4 cases with simulated patients and 2 paper cases). The cases are monitored and assessed by expert jurors. The evaluation is carried out on the basis of Entrustable Professional Activities (EPAs) [4] that have been applied in the tuition of medical students at the Münster Faculty of Medicine for four years. The competencies applicants will later have to display in order to pass the knowledge examination correspond to the competencies of students in their last year of study. Therefore, we use an evaluation scale in the course that was originally meant for the medical training. Based on different competencies (1-13 Core-EPAs) the applicants are attributed an entrustment level of 0-5 (corresponding excerpt from original Chen entrustment scale [5]):

- 0 – I would **not** allow this student **observe** this activity.
- 1 – I would allow this student **only to observe** this activity.
- 2 – I would allow this student to practice this activity under **complete direct supervision** or the **possibility of immediate intervention as coactivity with supervisor.**
- 3 – I would allow this student carry out this activity under **reactive/ on-demand supervision with supervisor immediately available and double-check of all findings.**
- 4 – I would allow this student carry out this activity under **remote supervision without supervisor direct available.**
- 5 – I would trust this student to carry out this activity **in full responsibility and to lead and supervise others doing this activity.**

**Figure 1:**
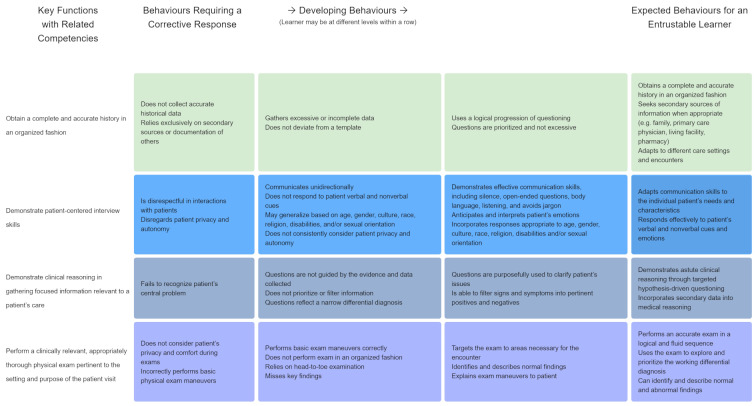
Assessment of the participants‘ competencies and the required degree of supervision based on the example of EPA 1 (Anamnesis and physical examination) in the assessment tool EPASS [6].

The entrustment levels correspond to the supervision degrees and comprise several sub-dimensions taking into consideration elements like skills and knowledge, as well as linguistic and interpersonal competencies (see figure 1 (Fig. 1)).

In order to pass the theoretical and practical parts of the examination and to attend the course applicants have to answer 50% of the questions correctly and achieve an entrustment level of >2 in the practical parcours test.

The course consists of 150 hours of in-class tuition and a preparatory stage using the Inverted Classroom model (160 hours). The in-class tuition consists of 10 two-day modules (Friday/Saturday) during which the major, most frequent and most relevant clinical pictures in the previously indicated examination subjects are discussed.

On Fridays, tuition takes place in small groups (6-7 participants). Each small group is supervised by expert tutors of the respective specialization. Clinically fitting patient cases are thematically studied in 75-minutes tuition units. In the afternoon specific examination techniques are rehearsed on models or reciprocally on a counterpart.

On Saturdays, typical illnesses are simulated by simulated patients. In rotation the small groups work on pre-prepared cases consistent with the respective module, with individual participants assuming the role of the interacting physician (anamnesis and physical examination) while the tutor watches the scene together with the other members of the group from an adjacent room. This interaction takes place in the Studienhospital Münster^®^ featuring “hospital rooms” or “consulting rooms” where cases can be simulated in a realistic environment and the participants’ interaction can be simultaneously monitored through two-way windows. 

Each small group is accompanied by a psychologist and the tutor. Participants have 10 minutes to take the anamnesis and make the physical examination.

The psychologist evaluates the participants on the basis of a Mini Clinical Evaluation Exercise (Mini-CEX) sheet. Equipped with tablets, the monitoring tutors are able to assess a participant’s competence and the required degree of supervision within a certain EPA. For an intuitive data input the web-based assessment tool EPASS was developed (see figure 1 (Fig. 1)). The collected data remains confidential and only serves to give the participants feedback on their professional development.

After each simulation scene two group members, the actor, the psychologist and the tutor each provide a comprehensive 360° feedback, with the actor focusing on his or her own sensibilities, thoughts, and emotions, as well as the demeanor of the counterpart and especially the facial expressions and gestures during the contact between physician and patient. In this context, linguistic or cultural aspects may certainly be of significance.

After the interaction each case is discussed for 20 minutes. In the middle and at the end of each course feedback is provided based on the Mini CEX-sheet. The participants do not receive their assessment of their entrustment level until end of the course.

#### 2.2. Case development and actor training

The cases are based on selected illnesses that have been specifically chosen and edited for the course, explicitly picking out realistic and frequently occurring settings that reflect reality in Germany. One week before their performance the actors receive an e-mail announcing the illness to be portrayed including an explanation and a detailed decryption of symptoms and some basic information about their role and the setting.

In contrast to the competence assessment for which it is essential that simulated situations can be exactly reproduced in order to have equitable test conditions, the simulations are not standardized during the course. Based on the specifications mentioned above the actors elaborate their role independently. In this way, they interact freely within the bounds of their own role biography according to the prediscribed symptoms and information. Directly before the performance, the actors can present their roles as they developed them and clarify any issues with the acting trainers.

#### 2.3. Implications of the COVID-19 pandemic on the course

The first course in 2020 was to take place in the manner described above from February 14 to April 25, 2020. Due to the pandemic and the ensuing restricting measures the course could no longer be held on-site with a total of 22 participants, four tutors, four psychologists and eight actors as planned. The face-to-face stage of the course which would originally have been held in class was converted one-to-one into a virtual seminar with conference rooms for small groups so that the course could take place online via Zoom^®^ without any missed dates.

In order to maintain the equivalence of the modules the most important cases of each module were discussed on Friday mornings based on power-point presentations. In the afternoon, the examination techniques were reviewed based on tutorials or power-point presentations. On Saturdays, selected cases were enacted by simulated patients and discussed. All participants, tutors and psychologists worked from their home office instead. Beforehand, all participants had been informed about the new framework conditions and had been trained in using the technology correctly.

##### 2.3.1. Instruction of participants and tutors

First, the participants received an operational training of how to use Zoom^®^ based on a specifically adapted manual. In a Zoom^®^-meeting tutors had been familiarized with the conference software and the didactics implementation.

##### 2.3.2. Training of the actors

Contact between physician and patient via telemedicine allows collaborative work without direct contact between the participants and the simulated patients. For technical reasons and reasons of role protection it had been decided that the actors would work contactless on site in the training hospital in dedicated rooms. The illness descriptions had been adapted and rewritten accordingly beforehand. In this framework it transpired that not all illnesses from our repertoire could be implemented via telemedicine. Simulations which for instance required make-up or made no sense without physical examinations had to be cancelled.

The actors were trained without contact via Zoom^®^. Later during the course, each of the simulated actors was allocated to a dedicated room in which the conference had already started. After a short briefing about the major functions of the conference software all procedures were conducted in the same way as in the “real” course.

##### 2.3.3 The exact sequences of the course – “Corona-Edition”

**Figure 2:**
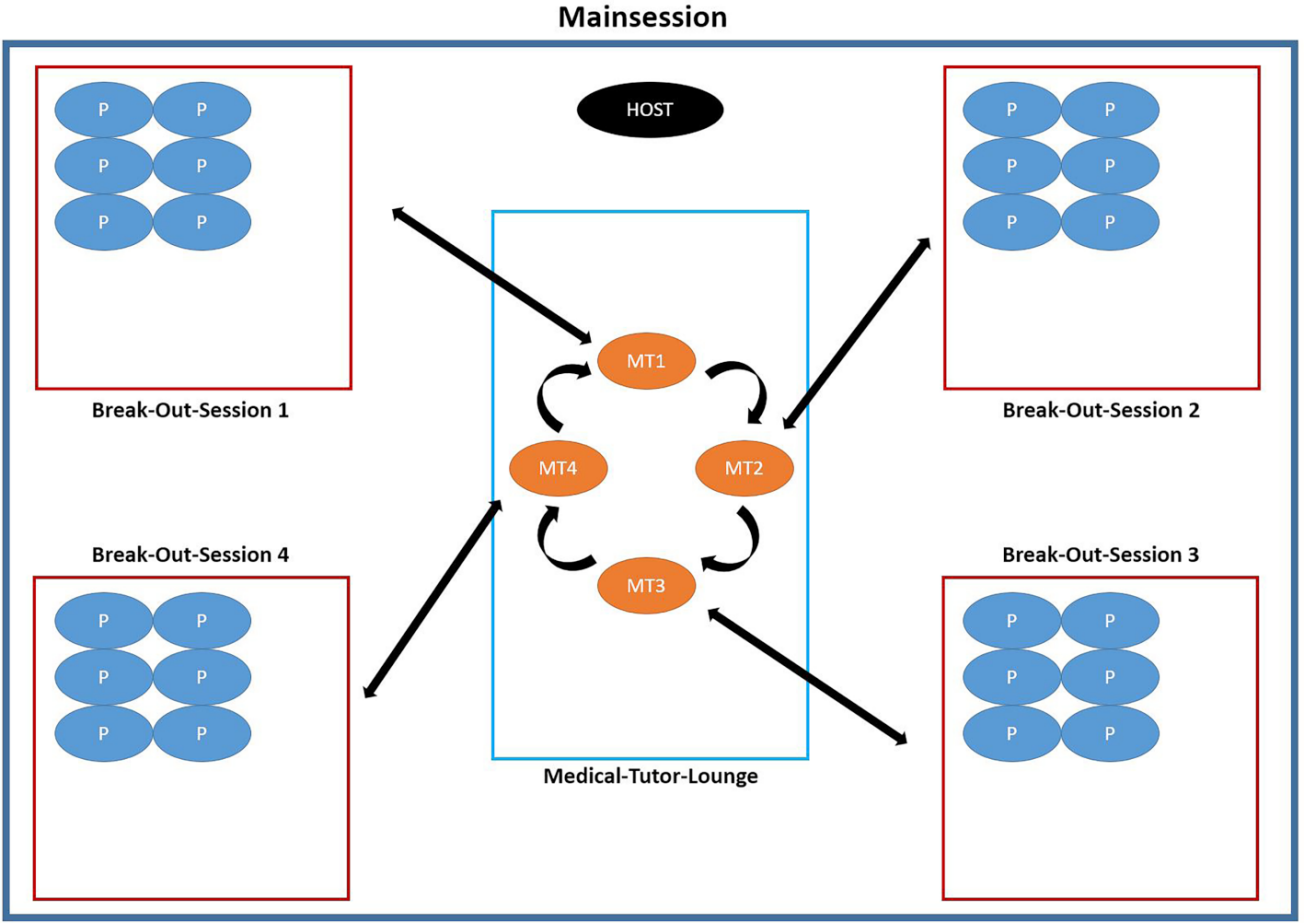
Procedure on Fridays: The host is in the main session. The tutors are trained in the virtual room and familiarized with the software. After that the tutors are allocated to the break-out sessions (group rooms). One tutor teaches a group of approx. 6-7 participants. 10 minutes before starting the participants join the meeting. The host allocates them to their respective small groups. The tuition starts. Over the whole course of time the host stays in the main session and is available for tutors and participants. At the changeover, only the tutors leave the break-out sessions and return to the main session where the host allocates them to the next group. The participants remain in their break-out session. All in all, there are five switches on a Friday. (P = participant; MT = medical tutor)

**Figure 3:**
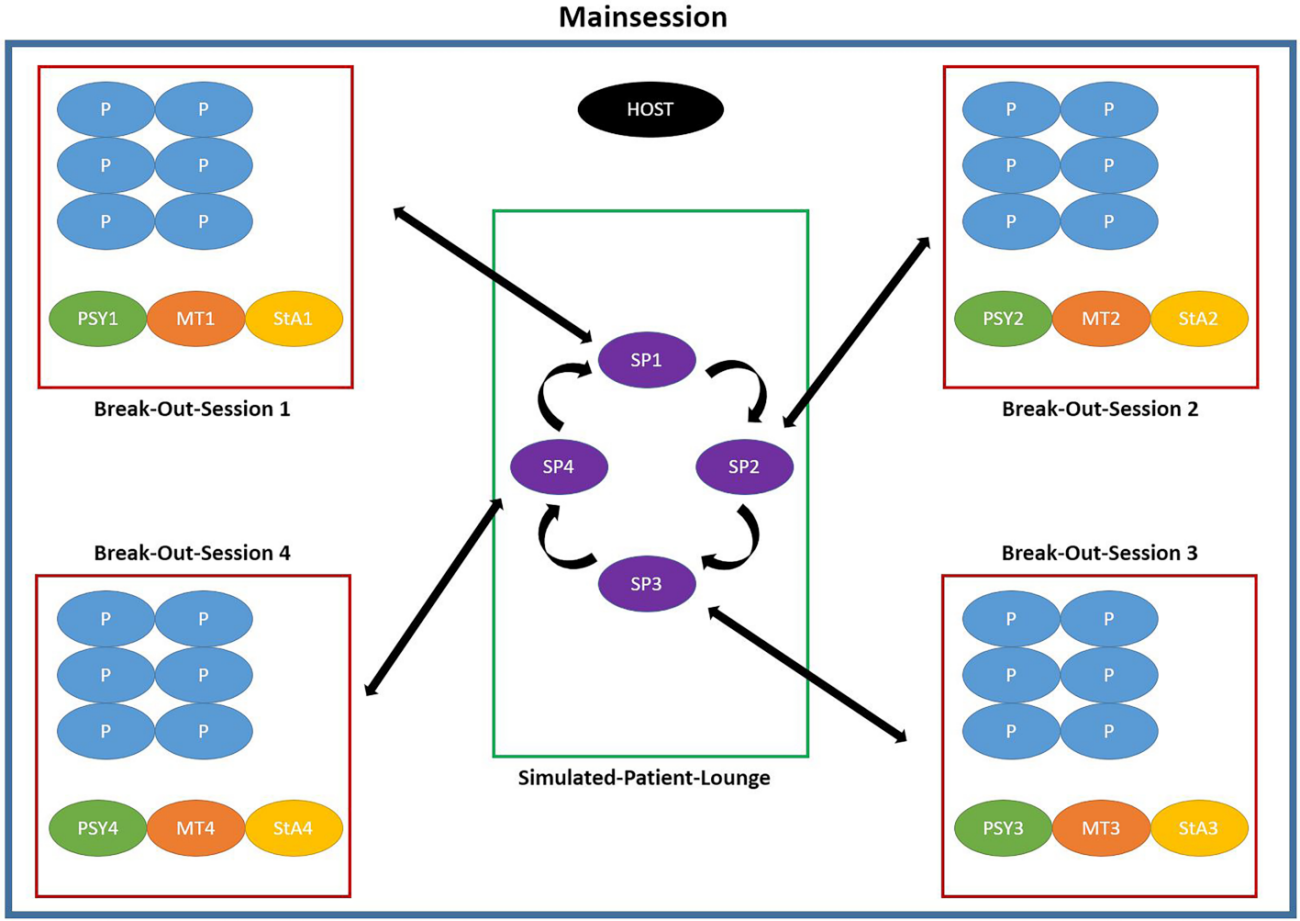
Procedure on Saturdays: The host is in the main session and expects the tutors who join him or her approx. 45 minutes before the planned start. In each break-out session there is one tutor and one psychologist to join each session. In the meantime, student assistants, actors and simulated patients join the meeting and are allocated to the respective break-out sessions. The student assistants use a split screen in order to display the assignments for the respective cases. 20 minutes before the start the participants join the meeting and are allocated to their break-out sessions. When the first case starts, the simulated patients are allocated to their respective break-out sessions. The host switches back to the main session and is now responsible for keeping the time schedule. The simulated patients have to leave their break-out session at a scheduled point of time. If they do not leave the room on time they are removed by the host. At the switch only the simulated patients leave the break-out sessions and tutors, psychologists and participants remain in their allocated break-out session. After the last case the student assistants, actor trainers and simulated patients each independently leave their break-out-session and all participants and tutors return to the main session. Then, the participants are seen off. There is a short final discussion with the tutors, then the host ends the meeting for all and the course day is terminated. (P = participant; PSY = psychologist; MT =medical tutor; StA = student assistant; SP =simulated patients)

40 minutes before the beginning of each module day, the host (organizer) starts the meeting and creates break-out sessions (group rooms and tutor or actor lounges), thereby mapping out the virtual work in small groups. The exact sequence of the course is shown in figure 2 (Fig. 2) and figure 3 (Fig. 3).

## 3. Results

The transformation of the course took place within the space of one week. Due to the quick implementation, the course could be conducted and completed uninterrupted within the intended time, despite the national lock-down measures. Five out of ten planned modules were carried out in class (until March 14, 2020) and five online (as from March 20, 2020). In total, the course thus consisted of 75 hours of in-class tuition and 75 hours of online tuition. The E-learning quota still amounts to 160 hours and has not changed due to the transition. None of the participants interrupted the course after its transition.

At the end of the course the participants reported that the teaching of expert knowledge in class as well as online worked out very well. What was described as problematic was the imparting of practical skills. Carrying out a physical examination via telemedicine was rated as unsatisfactory.

All in all, 7 out of 22 participants of the online-course (32%) underwent the knowledge examination and 6 (86%) passed the examination. In comparison to the 4 in-class courses which took place before, a total of 19 out of 72 participants underwent the knowledge examination (26%) and 18 (95%) passed the examination.

## 4. Discussion

At the end of the course the participants provided an oral evaluation and they clearly stated preference to in-class tuition. Despite adequate German language proficiency, participants from foreign countries still were hampered by a language barrier which additionally impedes communication in a setting of telemedicine. All participants perceived the course to be very helpful in the preparation for the knowledge examination.

We consider the lacking written evaluation as limitating. Our conclusions are merely based on the participants’ oral feedback. Moreover, the level of knowledge before and after the course and thus a possible gain was not explicitly measured.

The low number of participants that underwent the examination can be explained by the fact that the competent authorities sometimes take more than one year to allocate a date. Because of the COVID-19 pandemic this process took even longer. Despite the online format, however, course’s success rate did not deteriorate.

### 4.1. How did the participants perceive the transition?

#### 4.1.1. Organizer’s opinion

The transition to online tuition at short notice was challenging at first, but feasible with high commitment. This also included an increase of staff, securing of hard- and software, conversion of time schedules, elaborating new timelines and a good coordination within the team. 

##### 4.1.2. Tutors’ opinion

The transition to online tuition did not change the tutors’ work significantly. The case reviews on Fridays could easily be done online as most of the tutors had already prepared the cases as power-point presentations.

Explaining the physical examinations proved to be difficult. Despite tutorials and power-point presentations the teaching staff found imparting of practical skills to be unsatisfactory.

##### 4.1.3. Psychologists’ opinion

Before the COVID-19 pandemic, working together on weekends created a trustful learning atmosphere. It has been possible to highlight ethical, cultural and personal practices in the relationship between physician and patient and to reflect on them as colleagues. This was diminished in the online setting. Instead, issues connected with the demeanour of physicians in an online setting were rehearsed and reflected. Moreover, it soon became obvious that the camera worked like a “magnifying glass”: blunders in communicating with the patients often appeared over-large and more serious than they would be in live situations with a bigger focus.

##### 4.1.4. Opinion of the acting coaches

The transition from direct to virtual contact between the participants and the simulated patients was uncomplicated but required a lot of basic decisions and extra work. Although rewriting of illness descriptions and the preparation of the actors’ performances without contact was time-consuming it produced highly satisfactory results and contributed significantly to a successful virtual semester. In this framework, basic background decisions concerning the dramatic thrust and the role protection had to be made that have now become the basis of work with simulated patients via telemedicine in medical studies at the University of Münster.

##### 4.1.5. Participants’ opinion

For the participants the course was an opportunity to structure and expand their knowledge in the time leading up to the knowledge examination. Of particular importance was the training of communication between physician and patient. According to them, the transition to online tuition during the lockdown had been the best solution for them to prepare for the examination. If the course had been cancelled this would have meant a worse preparation for those who had to take the knowledge examination shortly.

## 5. Conclusion

The quick transition of the course from in-class tuition with simulated patient interaction to online tuition was feasible and successful. The experience gained in this context – especially in the transformation of tuition with the simulated patients – served as a blueprint for the successful implementation of the online-semester at the Faculty of Medicine of the Westfälische Wilhelms-Universität of Münster. It had not been previously known what options existed for synchronous live online tuition.

Following this course, online tuition with simulated patients has been complemented with external assessments based on the Core-EPAs. In the meantime, the training of the observers has also been optimized and an e-learning module as an inverted-classroom concept has been implemented.

It was soon noted during the course that online tuition is reaching its limits when it comes to imparting practical skills and that these tuition units will later be difficult to plan and to implement. These insights lead the faculty to develop solutions early, such as the use of tutorials to impart practical solutions, so that the summer semester 2020 could be conducted without any greater loss of tuition units. 

## First authorship

The authors Zornitsa Shomanova and Helmut Ahrens share the first authorship.

## Funding

This work was funded by the Federal Ministry of Labor and Social Affairs and the European Social Funds (Art. 8 (EG) Nr. 1828/2006) under the funding code 2019010337-11. 

## Acknowledgement

Thanks to Lukas Lohschelder for the technical layout.

## Competing interests

The authors declare that they have no competing interests.
